# The Use of Cardiac Magnetic Resonance Imaging in the Diagnostic Workup and Treatment of Atrial Fibrillation

**DOI:** 10.1155/2012/658937

**Published:** 2012-11-22

**Authors:** Peter Haemers, Piet Claus, Rik Willems

**Affiliations:** ^1^Division of Experimental Cardiology, Department of Cardiovascular Sciences, University of Leuven, B-3000 Leuven, Belgium; ^2^Division of Imaging and Cardiovascular Dynamics, Department of Cardiovascular Sciences, University of Leuven, B-3000 Leuven, Belgium; ^3^Division of Clinical Cardiology, Department of Cardiovascular Sciences, University of Leuven, Herestraat 49, B-3000 Leuven, Belgium

## Abstract

Atrial fibrillation (AF) is the most common cardiac arrhythmia and imposes a huge clinical and economic burden. AF is correlated with an increased morbidity and mortality, mainly due to stroke and heart failure. Cardiovascular imaging modalities, including echocardiography, computed tomography (CT), and cardiovascular magnetic resonance (CMR), play a central role in the workup and treatment of AF. One of the major advantages of CMR is the high contrast to noise ratio combined with good spatial and temporal resolution, without any radiation burden. This allows a detailed assessment of the structure and function of the left atrium (LA). Of particular interest is the ability to visualize the extent of LA wall injury. We provide a focused review of the value of CMR in identifying the underlying pathophysiological mechanisms of AF, its role in stroke prevention and in the guidance of radiofrequency catheter ablation. CMR is a promising technique that could add valuable information for therapeutic decision making in specific subpopulations with AF.

## 1. Introduction

Atrial fibrillation is the most common cardiac arrhythmia. The prevalence of AF is estimated at 1% in the general population and increases with age up to 10% in octogenarians [1]. Every adult older than 45 years has a 25% lifetime risk of developing AF [2]. The prevalence of AF is increasing and will reach epidemiological proportions that could pose a huge socioeconomical challenge [3]. AF is a progressive disease, which evolves from limited paroxysms to a persistent and sometimes permanent presence [4]. Structural, electrical and contractile remodeling processes underlying this progressive nature have been identified. The treatment of AF remains challenging and the longer AF exists the more difficult treatment becomes. Pulmonary vein ablation has emerged as a promising novel nonpharmacological treatment for AF. However, this is associated with significant complications, with little evidence of clear survival benefits [5]. 

Cardiovascular magnetic resonance imaging (CMR) is a noninvasive imaging technique which is used to assess the structure and function of the cardiovascular system. CMR is based on the same basic principles as magnetic resonance imaging (MRI), but with optimized features specific for cardiac imaging such as ECG gating and rapid imaging sequences. Different CMR sequences can be used to enhance the signal of the diseased tissue of interest. It is a very useful diagnostic tool and the therapy guidance's of various heart diseases. The major advantages of CMR are the absence of radiation exposure, the high temporal and spatial resolution, and the ability to characterize the composition of the tissue. However several disadvantages are also evident, such as cost, limited availability, incompatibility of certain prosthetic materials and time consuming analysis. In this paper we will focus on the possible use of CMR in AF. AF itself and the morphology of the atria impose additional obstacles for CMR. The complex geometry and the thin-walled anatomy make the structural analysis of the atria more challenging. Furthermore, as CMR depends heavily on ECG gating, the irregularity of the ventricular rhythm during AF can make its implementation problematic. 

However, CMR could improve ablation outcome by more effective preablation structure analysis, predicting the change of recurrence and the detection of postablation complications. Furthermore, CMR could help to reveal some of the underlying remodeling processes and could add important information, allowing more effective decisions on which patients would benefit most from anticoagulation. We will review the available scientific data on these possible advantages and challenges of CMR in the diagnostic workup and treatment of patients with AF.

## 2. Cardiovascular Magnetic Resonance and the Assessment of Left Atrial Remodeling

The knowledge of the pathophysiological mechanisms of AF has expanded rapidly the last decades [6, 7]. Atrial dilation can be found in the setting of diverse cardiac and pulmonary diseases and plays an important role in the development of AF. AF itself can also induce atrial dilation and in this way leads to the perseveration of AF [8, 9]. Measurements of left atrial (LA) dimensions play an important role in the workup of AF, since atrial enlargement is an important marker of LA structural remodeling and predictor of AF (re)occurrence and mortality [10]. B-mode or two-dimensional echocardiography is most commonly used in clinical practice to assess atrial and ventricular volume. However, echocardiographic volume measurements require correct angulations and two-dimensional based calculations of volumes depend on geometric assumptions about the LA shape. Three-dimensional echocardiography allows a more accurate LA volume assessment [11]. However, feasibility and correct interpretation may be more difficult during AF, due to the irregular heart rhythm, which results in a substantial variation of the LA volume. 

CMR has the advantage of providing an exact detailed image of the LA morphology, the pulmonary veins (PV), and the surrounding structures. Furthermore, CMR can assess the reservoir, conduit, and contractile function based on phasic changes in volumes [12]. A tight correlation between measured atrial volumes by CMR and true volumes measured in cadaveric casts has been observed [13]. Scanning during an irregular heart rhythm can result in a loss of image quality, which makes it sometimes necessary to repeat slices and to adjust the trigger window. However, the drawback of CMR to record every image slice during several heartbeats can be taken as an advantage since it would tend to compensate for the irregular heart rhythm during AF, allowing a more averaged LA volume. Therkelsen et al. demonstrated the feasibility of measuring atrial and ventricular volumes in AF patients with an irregular heart rhythm [14]. Several CMR studies demonstrated that in general patients with paroxysmal AF have larger LA volumes than control subjects [15, 16]. However, patients with paroxysmal AF without structural heart disease (“lone AF”) had no significant difference in atrial volumes compared with healthy volunteers [17]. CMR also demonstrated that the LA enlargement further increases when AF evolves from paroxysmal to persistent AF [18]. Therkelsen et al. compared atrial and ventricular volumes and ejection fraction (EF) between healthy volunteers and patients with persistent and permanent AF. The mean atrial volumes were similar between patients with persistent and permanent AF, but significantly larger compared to healthy volunteers. This suggests that LA dilation stabilizes when patients evolve from persistent to permanent AF [14]. 

CMR was also used to document the restoration of the function and structure of the atria and ventricles after cardioversion. Therkelsen et al. showed an immediate reversal of atrial systolic volumes and contractile function the day after cardioversion of patients with persistent AF. There was a further recovery of atrial dimensions and function at 30 days and 180 days. However, only the right atrial volumes were completely normalized 180 days after cardioversion. The restoration of ventricular function and dimensions started only 30 days after cardioversion and was incomplete at 180 days. These results suggest that structural remodeling of the atria and ventricles during AF could be permanent [19].

The pulmonary veins (PVs) play a critical role in the pathophysiology and treatment of AF [20]. CMR allows an accurate measurement of the PV dimensions and the branching pattern. However, identification of the true ostia remains problematic due to the lack of a clear anatomic border between the PVs and the LA. The PV size also varies significantly during the cardiac cycle [21]. Measuring the PVs in the sagittal plane at which the PVs separate from each other and the LA appears to be highly reproducible and may be advantageous for serial examinations [22]. Tsao et al. demonstrated significant dilation of the superior PVs with simultaneous LA dilation in paroxysmal and permanent AF. However, PV size couldn't predict the origin of arrhythmogenic trigger foci [23]. Similarly, Kato et al. have shown that patients with AF have larger PVs [15].

One of the unique features of CMR is the ability to characterize the tissue composition of the LA wall. Oakes et al. reported the feasibility to detect and quantify late gadolinium enhancement in the left atrium, assessed by delayed enhancement MRI (DE-MRI). They showed an association between regions of enhancement and low-voltage regions on electroanatomic maps. This suggests that late gadolinium enhancement may be a feasible way to detect LA fibrosis. They also identified two distinct patterns of enhancement: a more continuous pattern and a scattered pattern. The extent of LA wall enhancement was a significant predictor for the type of AF, with significant more LA enhancement in patients with persistent AF compared to paroxysmal AF [24]. Kuppahally et al. showed that the extent of LA enhancement on DE-MRI was inversely related with echocardiographic measured regional myocardial function, assessed with LA strain and strain rate [25].

## 3. Cardiovascular Magnetic Resonance and Stroke Management in AF Patients

Stroke is one of the most devastating complications of AF. However, thrombus formation in the LA is incompletely understood. The pathophysiological mechanisms can be summarized in the classic Virchow's triad: blood stasis, abnormal changes in the LA wall, and abnormal changes in blood constituents [26]. The left atrial appendage (LAA) is the suspected culprit in the majority of thrombo-embolic phenomena related to AF [27]. Transoesophageal echocardiography (TEE) is the clinical standard to evaluate thrombus formation in the LAA. However, TEE is semi-invasive and possesses a small risk of serious complications. Preliminary studies have shown that combined two-dimensional and three-dimensional transthoracic echocardiography (TTE) had comparable accuracy to two-dimensional TEE in evaluating the LAA for thrombus. However, a significant number of patients have a suboptimal acoustic window which limits the use of TTE evaluation of the LAA [28, 29]. CMR is an alternative, noninvasive tool, which allows a detailed evaluation of the LAA. Ohyama et al. showed that unenhanced CMR (without administration of contrast media) is a sensitive alternative for thrombus detection in the LAA. It has been shown that CMR can correctly differentiate thrombus from slow blood flow, appearing as spontaneous contrast in echocardiography [30]. However, in another study contrast-enhanced CMR (after the administration of contrast media) lacked diagnostic accuracy compared with the clinical reference TEE. It is postulated that the limited data acquisition time window in contrast-enhanced CMR resulted in insufficient spatial resolution precluding the accurate detection of LAA thrombus [31]. Caution should be taken in the use of CMR in clinical practice until the promising diagnostic accuracy of unenhanced CMR is confirmed in a larger multicenter study.

Stroke risk prediction is a key factor in the management of AF. This is crucial in the selection of patients which will benefit most from chronic anticoagulation therapy. There are several risk stratification schemes, of which the CHADS_2_ score is easy to apply and clinically well established [32]. However, the classic risk schemes have only a limited overall ability to predict thromboembolism, particularly in low-risk patients. Additional independent risk factors are needed to improve patient selection [33]. The more recent CHA_2_DS_2_VASc risk score takes additional clinically relevant nonmajor risk factors into account [34]. This approach leads to a better risk prediction in the patients with a CHADS_2_ score of 0 to 1 [35]. However, novel risk factors based on individual LA pathophysiological properties could further improve this risk stratification. CMR could help to identify some of the factors of the classic Virchow's triad. Beinart et al. showed a relation between stroke risk and larger LAA volumes, LAA depths, and necks. LAA neck dimensions remained predictive of stroke risk after adjustment for traditional stroke risk factors, indicating a possible role for its use in additional risk stratification [36]. Fyrenius et al. looked at the global flow patterns of the LA in healthy volunteers. They observed vortical flow in all subjects during systole and diastolic diastasis. This vortex formation may have beneficial effects in avoiding left atrial stasis and clot formation during sinus rhythm [37]. Further study is necessary to confirm loss of vortex formation during AF and it is relation with stroke risk. As suggested by Virchow's triad, structural changes to the LA wall may also contribute to the prothrombotic state in AF [26]. The extent of LA late gadolinium enhancement may be used as a marker for the severity of LA wall injury in AF. Daccarett et al. studied the association between LA late enhancement and the CHADS_2_ score. They found a clear association between patients with previous stroke and a higher percentage of LA late enhancement. This association was independent of all clinical stroke risk variables (CHAD score). However, it is unclear if this association was also independent from LA dilation [38]. As demonstrated by Fatema et al., there is a significant association between LA volume index, assessed by transthoracic echocardiography, and first-ever ischemic stroke [39]. Further research is necessary to evaluate which of these additional markers has the ability to substantially improve the predictive power of the current risk models. 

## 4. CMR to Guide Atrial Fibrillation Ablation

Haissaguerre et al. were the first to report that the pulmonary veins play an important role in the initiation of AF. They demonstrated that local radiofrequency catheter ablation of these ectopic beats could stop AF in the majority of patients [20]. However, recurrence rate was high and was associated with recurrent ectopic beats, indicating the need for a better mapping and ablation technique. As a result of leading-edge technologic advances, AF ablation has evolved into a safer and commonly performed procedure [40]. However, the success rate of AF ablation remains moderate, with a single-procedure success rate of 57% and multiple-procedure success rate of 71%, with a complication rate of 4.9% in a recent meta-analysis [5]. CMR could play an important role in the optimization of AF ablation, by accurate selection of candidates, improving the success rate of the procedure and reducing the chance of complications.

### 4.1. The Role of Preablation CMR

Preprocedural CMR can be used as a non-invasive imaging tool to delineate the relevant anatomical structures and to assess the parameters which are predictive for recurrent AF.

Electrical isolation of the PV is the cornerstone in AF ablation. This requires a detailed regional anatomic visualisation before the ablation procedure. The integration of pre-procedural anatomic information and electroanatomic mapping is associated with superior procedural success and safety [41]. A comparison of CMR with CT showed similar details which allowed effective evaluation of the PV anatomy [42]. A study by Kato et al. demonstrated that 38% of the AF patients have a variant PV anatomy [15]. Similar results were observed by Anselmino et al. where only 40% of the patients had a typical PV branching pattern (2 left and 2 right PVs). The most frequent variant branching patterns are a common left trunk and an additional right middle PV [18].

Besides the identification of the PV branching pattern and LA morphology, CMR can also assess parameters predictive for recurrent AF after ablation. Several CMR studies have identified various potential predictive parameters, such as LA volume, extent and pattern of LA wall late gadolinium enhancement, and pericardial fat. LA volume was an independent predictor for recurrence after ablation in a mixed group of patients with paroxysmal and persistent AF [43]. However, LA volume couldn't predict the success of ablation in patients with exclusive paroxysmal AF [44]. Oakes et al. showed that the extent of LA wall enhancement was the most significant predictor for the success of AF ablation. Furthermore, the location of late enhancement appeared to predict the success. Success was higher when late enhancement was limited primarily to the posterior wall and septum. Also LA volume was predictive for recurrent AF, although the extent of LA wall enhancement had a greater adjusted odds ratio [24]. Another interesting finding by Wong et al. is the association between pericardial fat and presence of AF, severity of AF, LA volume, and poorer outcomes after AF ablation. However, the study design did not allow any conclusions to be drawn on causality [45].

### 4.2. The Role of Postablation CMR

A particular strength of CMR in the post-procedural period is the ability to visualize scar formation. CMR can also be used to study the effects of ablation on LA structure and function, and to detect PV stenosis.

Several investigators demonstrated the feasibility to assess postablation scar with DE-MRI [46–48]. McGann et al. detected hyperenhancing and nonenhancing lesions. The nonenhancing lesions demonstrated no-reflow characteristics and were a better predictor for scar formation at 3 months [46]. A correlation between procedure outcome and the extent of scar formation has also been described. Patients with minimal scar formation had a higher rate of AF recurrences [47]. Furthermore, visualization of postablation scar can detect incomplete isolation and thus can be useful in assessing the reason for failure. Moreover, detection of the location of the isolation gaps can be helpful in planning a redo procedure [47].

Radiofrequency ablation results in a significant decrease of the LA size. However, a similar decrease in LA size was noted in patients with a successful ablation as in patients with AF recurrence. These data suggest that the reduction in LA size may be induced by the ablation procedure itself, rather than reverse remodeling [49]. Nori et al. studied the effects of ablation on global and regional LA function. Global LA transport function and regional LA motion were decreased 3 months following ablation in patients with paroxysmal AF. However, in patients with persistent AF, global, and regional functions were improved. Here, the positive reverse remodeling due to restoration of sinus rhythm seemed to outweigh the negative effects of the ablation procedure [16]. It was also demonstrated by Wylie Jr. et al. that the extent of LA scar formation influences the atrial systolic function after ablation, with a more pronounced decrease in LA systolic function in extensive scar formation [48]. AF ablation also influence the PVs. Tsao et al. noted a reduction of the ostial area of the superior PVs after successful ablation, as well as a geometric adaptation towards a rounder shape of the ostia. In patients with AF recurrence there was further LA and PV enlargement [50]. 

AF ablation can induce unwanted and harmful effects on the PVs. Case reports of PV stenosis with severe pulmonary hypertension appeared shortly after the introduction of catheter ablation of AF [51]. CMR allows sequential PV analysis without repeated radiation exposure and is comparable to radiographic angiography for the detection of PV stenosis [52]. Dong et al. reported a ≥3 mm PV narrowing in 38% of PVs 8–10 weeks following ablation. However, moderate (50–70%) and severe (>70%) stenosis was only noted in 3.2 and 0.6%, respectively [53]. Distal ablations inside the PV, individual PV encircling lesions, and larger PV size are all associated with a higher risk of stenosis [53, 54].

## 5. Summary

Atrial fibrillation is a very frequent disorder, with an underlying continuously evolving atrial substrate. Detailed imaging of the LA, PVs, and surrounding structures during AF progression is crucial for good patient management. CMR has multiple advantages over other imaging modalities. This allows a detailed assessment of the LA morphology and function and is currently the only technique which allows an appraisal of the extent of LA wall injury. However, cost and time will limit routine use of CMR in clinical practice. Many of these techniques are new and need to be confirmed in larger multicenter studies. Nevertheless, it is clear that CMR can play an important role in specific AF patient subpopulations, such as patients undergoing pulmonary vein ablation.
